# Effectiveness of Robot Care Intervention and Maintenance for People with Dementia: A Systematic Review and Meta-Analysis

**DOI:** 10.1093/geroni/igae110

**Published:** 2024-12-21

**Authors:** Su-Jung Nam, Eun-Young Park

**Affiliations:** Department of Consumer Sciences, Convergence Program for Social Innovation, College of Social Sciences, Sungkyunkwan University, Seoul, Republic of Korea; Department of Secondary Special Education, College of Education, Jeonju University, Jeonju, Republic of Korea

**Keywords:** Effect size, Outcomes, Robot type

## Abstract

**Background and Objectives:**

Robots have the potential to improve the quality of life of people with dementia. This study examined the effectiveness of robot care intervention and maintenance effect for people with dementia.

**Research Design and Methods:**

Meta-analytical procedures were used to identify and synthesize articles for analysis. Coding procedures were used to record the moderators, including robot type, outcomes, intervention length, intervention duration, and intervention frequency. Hedge’s *g* statistic was employed to interpret effect sizes and quantify individual research findings.

**Results:**

The literature review identified 20 eligible randomized controlled trials. Meta-analysis results indicated an overall small effect of *g* = 0.286 for robot care intervention and *g* = 0.279 for robot care maintenance. Outcomes for robot care intervention indicated a small and significant effect size at *g* >0.2, whereas the Bomy robot type had an insignificant effect size. Outcomes for robot care maintenance showed a medium and significant effect size.

**Discussion and Implications:**

This study confirmed the intervention effect of robot care on people with dementia and its sustainability for neuropsychiatric and social health outcomes. This highlights the effectiveness of humanoid-type robots in dementia care.

**Translational Significance:** People with dementia often experience challenges that negatively impact their social health, such as decreased social participation and increased health care costs. This study found that using robotic care interventions significantly improved social health outcomes, including enhanced appraisal and social participation. These positive effects were sustained over time. Integrating robotic care into dementia care practices can lead to substantial improvements in social health and reduce neuropsychiatric outcomes.

## Background and Objectives

Dementia currently affects 46.8 million individuals globally, and this figure is projected to surge significantly to 131.5 million by 2050 (World Health Organization, 2012). According to Alzheimer’s Disease International, the global economic burden of dementia in 2010 reached US$604 billion, exceeding 1% of the global gross domestic product; this worldwide cost was anticipated to increase to $1 trillion by 2018 (Petersen et al., 2017). Over the past two decades, new research and care practices have highlighted how individuals cognitively, emotionally, and socially adapt to the changes brought about by dementia, as well as how various interventions can help those with dementia and their caregivers maintain a positive sense of well-being (Clare et al., 2014; Droes et al., 2011; Nygard, 2004; Van’t Leven et al., 2013).

In recent years, the increasing number of older adults with chronic conditions has highlighted the need for adaptive and self-management skills, extending beyond World Health Organization’s definition of health as “a state of complete physical, mental, and social well-being.” Physical health refers to the ability to maintain physiological homeostasis in a changing environment, whereas mental health is defined as a sense of coherence that supports successful coping with and recovery from psychological stress. Social health is characterized by three dimensions: (1) the ability to fulfill one’s potential and obligations, (2) the ability to manage life with a degree of independence despite illness, and (3) the ability to participate in social activities, including work (Huber et al., 2011). In addition, Van der Roest et al. (2009) found that aside from support for memory issues, one of the most common unmet needs among people with dementia is the desire for social health. As is the case with the general population, social health significantly influences the quality of life and mortality rates in patients with dementia (Gitlin et al., 2006, 2009; O’Rourke et al., 2015). Therefore, the social health of people with dementia has received as much attention as cognitive health, and addressing this issue is important for optimizing the quality of life of people with dementia. Growing evidence suggests that psychosocial interventions that promote social relationships can improve the well-being of people with dementia (Jang et al., 2004; van Dijk et al., 2012). Therefore, the social health aspect that encompasses a patient’s entire life must be addressed in conjunction with cognitive therapy to effectively manage dementia.

In a study examining social health in the context of dementia, social health was categorized into individual and socioenvironmental dimensions (Vernooij-Dassen et al. 2022). The individual dimension includes capacities (e.g., reciprocity), independence (e.g., autonomy), and social participation (e.g., social engagement, social leisure activity, and social isolation). The socioenvironmental dimension encompasses structure (i.e., frequency of contact, social network, living alone, and being married), function (i.e., inability to help, exchanging support), and appraisal (i.e., loneliness). The relationship between social health, decline in cognitive function, and the development of dementia has been extensively proposed (Lenart-Bugla et al., 2022;). Existing research has suggested the need to prioritize both medical treatment and the maintenance of social health to enhance the quality of life and alleviate cognitive decline in people with dementia (Bennett, Schneider, Tang, Arnold, & Wilson, 2006).

Interest in promoting social health through technological interventions has increased steadily in recent years. The burgeoning field of robotics is garnering attention as a promising domain for assisting individuals with dementia (Khosla et al., 2021). The overwhelming consensus derived from these studies is that robotic interventions significantly contribute to the mitigation of stress and loneliness, augmenting engagement, mood, and overall well-being of older adults with dementia. Robotics technology is advancing rapidly, particularly in artificial intelligence systems aimed at enhancing human relationships. One area in which this progress has been particularly impactful is dementia care, wherein robotic interventions have shown great promise. These robots are effective in reducing negative emotions and behavioral symptoms, while simultaneously improving patient engagement and overall care quality in a cost-effective manner (Jung et al., 2017; Mervin et al., 2018). Additionally, they provide significant psychological and physiological benefits, such as reducing symptoms of depression, promoting communication, and encouraging social interaction (Bemelmans et al., 2012). Studies have demonstrated that robotic care interventions improve the quality of life and neuropsychiatric status of people with dementia (Jøranson et al. 2021; Moyle et al., 2017).

However, these investigations exhibit notable limitations, including a cross-sectional design devoid of a baseline evaluation, a constrained sample size (11–25 participants), and predominantly laboratory-centric robot–human interactions conducted within the confines of group sessions characterized by delimited temporal extents (25–60 min) (Hebesberger, Koertner, Gisinger, & Pripfl, 2017; Moyle, Jones, Dwan, Ownsworth, & Sung, 2019; Pino, Boulay, Jouen, & Rigaud, 2015). Another limitation of robot care research is the relative neglect of robot types. Within the domain of psychosocial interventions, the evaluation of PARO, a therapeutic pet robot, is underway (Chen et al. 2024; Jøranson et al., 2015; Liang et al., 2017; Mervin et al., 2018; Moyle et al., 2013; Petersen et al., 2017; Pu, Moyle, Jones, & Todorovic, 2021; Robinson et al., 2013; Thodberg, Sørensen, Christensen, et al., 2016). Nevertheless, research outcomes regarding the impact of diverse robot types, such as humanoid robots (Chen, Lou, Tan, Wai, & Chan, 2020a; Park, Jung, & Lee, 2021; Zhou, Barakova, An, & Rauterberg, 2021), are limited. Furthermore, the effects associated with different robot types have not been comparatively analyzed.

Accordingly, the current study aims to synthesize past research on robot care intervention and maintenance effect for people with dementia. It addresses the following research questions: (a) What are the characteristics of the included studies? (b) What are the overall effects of robot care intervention and maintenance for people with dementia? (c) Which variables, namely, robot type and outcomes, moderate the outcomes of robot care? (d) What are the moderator effects of intervention length, duration, and frequency on the effects of robot care intervention?

## Research Design and Methods

### Article Search Procedures

A comprehensive literature search was conducted using the electronic databases of EBSCOhost, Web of Science, CHINAL, and PubMed to identify studies that met the inclusion criteria. The search was performed using several keywords in various combinations. The search included all papers published before August 31, 2023. Exactly match quotation marks were used for terms that included more than one word (Supplementary Material Section 1).

### Article Selection Criteria

The inclusion criteria were studies that: (a) were published in peer-reviewed journals, (b) were written in English, (c) included participants with dementia, (c) included social health as a dependent measure, (d) implemented and collected data on robot care, and (e) employed a randomized control treatment design. Supplementary Figure 1 presents the specific study selection process.

The exclusion criteria were studies that: (a) were not related to dementia, (b) did not involve any intervention, (c) were not randomized controlled trials, (d) had no data for calculating effect sizes, and (e) were not focused on robotic care.

### Methodological Quality Assessment of Selected Articles

One researcher and one assistant independently applied the Cochrane Risk of Bias 2 (RoB 2) tool to assess the methodological quality of the included randomized controlled trials (RCTs) and the potential for bias of each included study design (Sterne et al., 2019). The RoB 2 tool assesses five domains: (a) risk of bias arising from randomization, (b) risk of bias due to deviations from intended intervention, (c) risk of bias due to missing outcome data, (d) risk of bias due to outcome measurement, and (e) risk of bias due to the selection of reported results. All included studies were independently assessed, and the risk of bias for each study was classified as “low risk,” “some concerns,” or “high risk.” Discrepancies in the ratings were subjected to discussions between researchers. The results of the quality assessment did not affect the inclusion or exclusion criteria for the studies analyzed.

### Variable Coding

Articles that met the inclusion criteria were coded using a researcher-developed spreadsheet. This coding sheet comprised three parts. The first part coded related variables. Thirteen variables were coded: author (year), number of participants, number of female participants, number of male participants, age, key inclusion criteria, dementia severity, intervention length, intervention duration, intervention frequency, robot type (PARO, humanoid, or Bomy), outcome (neuropsychiatric, social participation, and emotional/appraisal), and maintenance status. PARO, a baby harp seal robot, is a type of social robot designed for pet therapy for people with dementia (Shibata et al., 2004). Bomy, developed by Robocare (2019), provides cognitive training and day care for the elderly. Bomy has a gray body with two blue, oval-shaped “eyes” displayed on a black screen, resembling a face. The neuropsychiatric outcomes of the included studies were coded. Outcomes such as activity, engagement, and social interaction were coded as social participation. Outcomes such as anger, sadness, and pleasure were coded as appraisal. As the primary focus of this meta-analysis was social health, outcomes such as cognition, blood pressure, and medication were excluded from the analysis. The second part of the coding sheet was used for quality assessment. The third part captured the data required to calculate effect sizes: number of participants in the experimental and control groups, pre- and post-test scores, standard deviations in the experimental and control groups, and *t*, *F*, and *p* values.

Prior to coding each study, the coders underwent a one-hour training session covering the definitions of the coding variables. They also practiced data coding by using a coding spreadsheet. Following the training, each coder independently coded all the articles twice. The average inter-coder reliability was 98.7%. Any disagreements were resolved through discussions among the coders.

### Effect Size Calculation

In this meta-analysis, the unit of analysis was the total effect size estimation. Effect sizes were derived by converting the standardized mean differences to Hedge’s *g* values (Bornstein et al., 2021).

The formula for estimating Hedge’s *g* is:


$$\begin{array}{l} g\;\;\;=\;\;\;J\;\;\;\text{x}\;\;\;d \end{array}$$

where Cohen’s *d* is calculated as the difference in means divided by the pooled standard deviation, and *J* is the correction factor for small sample sizes, given by


$$\begin{array}{l} J=1-\frac{3}{4\;\left(N-2\right)-1} \end{array}$$

where *N* is the total sample size (sum of both groups).

Hedge’s *g* values were interpreted according to guidelines provided by Cohen (1988). Values between 0.2 and 0.5 are regarded as small, those between 0.5 and 0.8 as medium, and values above 0.8 as large. Various tools can be used to evaluate different effects; for instance, a standardized mean difference can serve as a criterion for comparing studies (Borenstein, Hedges, Higgins, & Rothstein, 2021). In the current study, the effect size and variance of each individual study were computed and integrated using suitable formulae. The effect of robotic interventions was determined by calculating the effect sizes from twenty studies, whereas the effect on maintenance was assessed through effect size calculations from seven studies.

### Heterogeneity Test

The effect sizes reported by each study were assessed in terms of whether they stem from a homogeneous or heterogeneous population. The appropriate analysis model was then selected. Two main models were used to integrate effect sizes into the meta-analysis: the fixed effects model, which assumes homogeneity within the population; and the random effects model, which accounts for the variation between studies. The statistical *Q*-test was used to assess the degree of sample heterogeneity, with *p*-values indicating the extent of variability between studies (Higgins & Thompson, 2002).

### Outlier Analysis

The effect size for each study was computed after eliminating statistical anomalies in the synthesis of the effect sizes of the individual studies. The effect sizes associated with the extreme mean differences were initially examined for outlier status and excluded from the aggregation process if deemed outliers (Huffcutt & Arthur, 1995). To confirm the presence of outliers, we confirmed the residuals through a basic analysis of the total effect of robot care intervention. The threshold for identifying outlier residuals was set to 2.70 (*p* < .01). No outliers were detected in the included studies.

### Publication Bias

Publication bias can lead to overestimation of meta-analysis results (Rosenberg, 2005). To validate the results of the meta-analysis, we employed two methods for verifying bias: fail-safe *N* and Egger’s test (Egger et al., 1997). Fail-safe *N* refers to the number of missing studies that would nullify the observed effect.

### Moderator Analysis and Meta-Regression

We conducted a moderator analysis to identify potential variables that may influence the outcomes of robot care intervention. Categorical variables were examined using meta-one-way analysis of variance (ANOVA) whereas continuous variables were analyzed using meta-regression (Borenstein et al., 2021). Meta-regression involves regressing the effect sizes on continuous study characteristics to assess whether the moderator influences the effect size (Huizenga, van Bers, Plat, van den Wildenberg, & van der Molen, 2009). In the meta-ANOVA, the unit of analysis was the individual effect size (Cooper, Hedges, & Valentine, 2009). We employed meta-ANOVA to investigate differences between dependent variables and robot types, and meta-regression to analyze factors such as intervention length, duration, and frequency. Comprehensive Meta-Analysis software (version 4.0) was used for the analysis (Borenstein et al., 2021).

## Results

### General Characteristics of Individual Studies

The characteristics of individual studies are presented in Table 1. A total of 1,672 people with dementia participated in the 20 studies reviewed in this meta-analysis. The participants were aged over 60 years. The most frequently used robot type was PARO. Sixteen studies (80.0%) implemented interventions with PARO, three (15.0%) used humanoid robots, and only one (5.0%) used Bomy. The intervention duration ranged from 10 min to 60 min. Two studies (10.0%) had a free intervention duration whereas one (5.0%) had a flexible duration. One study did not report information regarding duration. Length ranged from 4 weeks to 32 weeks. Seven studies (35.0%) performed interventions twice per week whereas six studies (30.0%) performed interventions thrice per week. Two studies (10.0%) did not report frequency. Most studies were implemented in centers, facilities, and nursing homes. Only one study was conducted in a hospital setting. Seven studies reported the results of intervention maintenance. Three studies (15.0%) reported all three outcome categories, six (30.0%) reported only social participation outcomes, two (10.0%) reported only appraisal outcomes, two reported only neuropsychiatric outcomes, and six studies reported two of three outcomes.

**Table 1.** Characteristics of Individual Studies

Study	Sample size; female (% or *n*)	Participants		Intervention				Setting	Outcomes	Maintain	Quality rating				
		Age (yr)	Dementia severity	Type	Length (min)	Duration (weeks)	Frequency (per week)				D1	D2	D3	D4	D5
Liang et al. (2017)	24; 64%	67-98	NR	PARO	30	6	2~3	Day care center in NZ	Neuropsychiatric Social participation Appraisal	R	S	L	L	S	S
Jøranson et al. (2016)	53; 70%	83.9	Mid = 7.4% Moderate = 48.1% Severe = 44.4%	PARO	12	30	2	Nursing home in Eastern Norway	Social participation	R	S	L	L	L	L
Chen et al. (2020a)	103; 40	87	MoCA-5: 6.2 NPI-Q: 2.8	humanoid robot (Kabochan)	Free	32	7	Long-term care facilities in HK	Social participation	NR	L	L	L	S	L
Chen et al. (2020b)	103; 40	87	MoCA-5: 6.2 NPI-Q: 2.8	humanoid robot (Kabochan)	Free	32	7	Long-term care facilities in HK	Neuropsychiatric Social participation Appraisal	NR	S	L	L	S	S
Feng et al. (2020)	21; 5	86.6	mild: 1 middle: 2 middle to severe: 3 severe: 1	PARO	10	20	NR	Vitalis living facility in the Netherlands	Social participation Appraisal	NR	S	L	L	L	S
Moyle et al. (2013)	36; NR	NR	mid to late stage	PARO	45	5	3	Residential care facility in Australia	Neuropsychiatric Social participation Appraisal	NR	L	L	L	L	S
Bradwell et al. (2022)	63; 22	87.14	NPI: 30.80	PARO	Flexible	16	NR	Care homes in UK	Neuropsychiatric Social participation	NR	L	L	L	L	S
Valentí Soler et al. (2015)	71; 88%	84.68	GDS 4 = 2% GDS 5 = 17% GMS 6 = 44% GDS 7 = 37%	PARO	35	12	2	Public nursing home and day care center	Neuropsychiatric	NR	S	L	L	L	S
Mervin et al. (2018)	278; NR	over 60	MMSE: 7.71	PARO	15	10	3	Facilities in Australia	Neuropsychiatric	R	S	L	L	L	S
Pu et al. (2021)	43; 18	86.48	NR	PARO	30	6	7	Residential aged care facilities in Australia	Social participation	R	S	L	L	L	S
Park et al. (2021)	135; 98	75.5	NR	humanoid Robot (Sil-Bot)	60	6	2	S-City dementia center in SK	Appraisal	NR	L	L	L	L	S
Chen et al. (2024)	52; 17	81.81	MMSE = 22.04	PARO	60	8	3	Long-term care facilities in Taiwan	Appraisal Social participation	NR	S	L	L	L	S
Moyle et al. (2018)	122; 53	84	RUDAS = 7.6	PARO	15	10	3	Long-term care facilities in Australia	Social participation	R	L	L	L	L	L
Jøranson et al. (2015)	53; 70%	83.9	CDR: 7.4% mild; 48.1% moderate; 44.4% severe	PARO	30	12	2	Nursing home in Norway	Neuropsychiatric Appraisal	R	L	L	L	L	S
Thodberg, Sørensen, Christensen et al. (2016)	70; 69%	85.5	NR	PARO	10	6	2	Nursing home in Denmark	Social participation	R	S	L	L	L	S
Robinson et al. (2013)	40; 27	55-100	NR	PARO	NR	12	2	Retirement home in NZ	Appraisal Social participation	NR	S	L	L	L	S
Petersen et al. (2017)	61; 27	83.5	NR	PARO	20	12	3	Facilities	Neuropsychiatric Appraisal	NR	L	L	L	L	S
Moyle et al. (2017)	275; 101	84	RUDAS = 6.5	PARO	15	10	3	Long-term care facilities in Australia	Neuropsychiatric Social participation Appraisal	NR	L	L	L	L	L
Thodberg, Sørensen, Videbech et al. (2016)	60; 19	83.9	Mild/moderate = 60	PARO	10	6	2	Nursing homes in Denmark	Social participation	NR	L	L	L	L	L
Lee et al. (2020)	54; 9	73.6	Mild cognitive impairment	Bomy	60	4	5	Hospital in SK	Appraisal	NR	L	L	L	L	L

*Note*: NR = not reported, R = Reported; MoCA = Montreal Cognitive Assessment; NPI-Q = Neuropsychiatric Inventory Questionnaire; GDS = Global Deterioration Scale; MMSE = Mini-Mental State Exam; RUDAS = Rowland Universal Dementia Assessment; CDR = Clinical Dementia Rating; NZ = New Zealand; HK = Hong Kong; UK = United Kingdom; SK = South Korea; D1 = Randomization process; D2 = Deviations from the intended interventions; D3 = missing outcome data; D4 = measurement of the outcome; D5 = selection of the reported result; L = low risk; S = some concerns; H = high risk.

### Publication bias

Rosenthal suggested that the criterion for evaluating publication bias is five times the number of studies plus 10. In this study, with 20 articles analyzed, the threshold was set at 110, and a robustness index above this value indicated no publication bias. The analysis revealed a robustness index of 952, strongly suggesting the absence of publication bias. Additionally, Egger’s regression intercept was not significant (*p* = .21), further confirming that the findings were not influenced by publication bias.

### Quality of studies

Of the 20 included studies, 10 (50%) provided detailed information on the randomization process and were considered low risk. Ten studies (50%) did not describe the allocation sequence concealment under randomization to reduce selection bias. None of the studies showed deviations from the intended interventions or missing outcome data. Only three studies showed some concerns about outcome measurement because they did not describe the blinding of the outcome assessors and their outcome assessment could have been influenced by the knowledge of the intervention received (Chen et al., 2020a; Chen, Lou, Tan, Wai, & Chan, 2020b; Liang et al., 2017). Six studies showed a low risk regarding the selection of the reported results (Chen et al., 2020a; Jøranson et al., 2016; Lee et al., 2020; Moyle et al., 2017, 2018; Thodberg, Sørensen, Videbech, et al., 2016).

### Overall effects

The results of the homogeneity test for robot care intervention were significant (*Q* = 34.694, *df* = 19, *p* < .05), and a random effects model was employed to aggregate the effect size. The results of the homogeneity test for intervention maintenance were not significant (*Q* = 5.851, *df* = 6, *p* > .05), and a fixed effects model was employed to aggregate the effect size (Supplementary Table 1).

**Figure 1.**
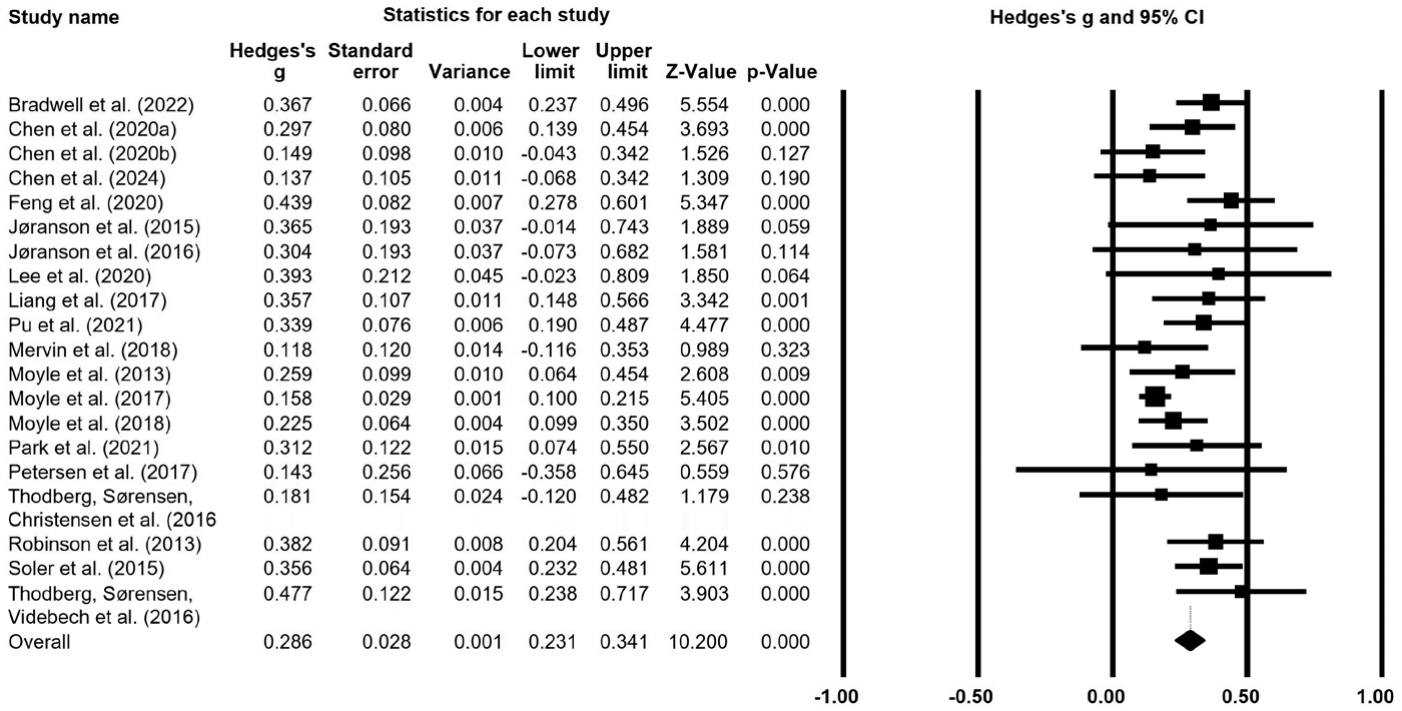
Forest plot of robot care intervention.

Figure 1 shows the effectiveness of robot care intervention reported by each study. Twenty effect sizes for robot care intervention were calculated. The effect sizes in eight studies were not significant (Chen et al., 2020b, Chen et al. 2024; Jøranson et al., 2015, 2016; Lee et al., 2020; Mervin et al., 2018; Petersen et al., 2017; Thodberg, Sørensen, Christensen et al., 2016) whereas those in 12 studies were significant. The overall effect sizes for robot care intervention were medium (*g* = 0.286), and the individual effect sizes were small.

Seven effect sizes for intervention maintenance were calculated (Supplementary Figure 2). The effect sizes of two studies were not significant (Liang et al., 2017; Thodberg, Sørensen, Christensen, et al., 2016) whereas those of five studies were significant. The overall effect sizes for intervention maintenance were small (*g* = 0.279).

### Subgroup Analysis

Table 2 presents the effect sizes of robot care intervention according to outcomes. These effect sizes were 0.208 (95% CI = 0.148–0.267) for appraisal, 0.258 (95% CI = 0.192–0.324) for neuropsychiatric outcome, and 0.287 (95% CI = 0.231–0.342) for social participation. The meta-ANOVA test indicated that the difference in effect sizes according to outcomes was not statistically significant (*Q* = 3.649, *df* = 2, *p* = .161).

**Table 2.** Effect Sizes of Intervention and Maintenance by Outcomes

Category	Moderators	*k*	95% CI	ES	+95% CI	SE	*Q*	*p*
Intervention	Outcomes							
	Appraisal	48	0.148	0.208	0.267	0.001	32.978	.940
	Neuropsychiatric	42	0.192	0.258	0.324	0.001	32.432	.828
	Social participation	84	0.231	0.287	0.342	0.001	54.875	.993
	Total between						3.649	.161
	Type of robot							
	Bomy	2	-0.023	0.393	0.809	0.212	0.124	.725
	humanoid	17	0.181	0.280	0.378	0.050	8.286	.940
	PARO	155	0.210	0.247	0.284	0.019	114.719	.992
	Total between						0.806	.668
Maintenance	Outcomes							
	Appraisal	2	0.131	0.579	1.027	0.052	0.650	.420
	Neuropsychiatric	5	0.164	0.318	0.471	0.006	3.752	.441
	Social participation	30	0.182	0.260	0.337	0.002	22.541	.797
	Total between						2.205	.332

*Note*: *k = *number of effect size; CI = confidence interval; ES = effect size; SE* = *standard error.

The effect sizes according to robot type ranged from 0.247 to 0.393. The effect size of the Bomy intervention was not significant while that of the humanoid intervention was significant and greater than that of PARO.

The effect sizes of intervention maintenance according to outcomes ranged from 0.260 to 0.579. The meta-ANOVA test indicated that the difference in these effect sizes was not statistically significant (*Q* = 2.205, *df* = 2, *p* = .332). The effect size for appraisal was the largest among all outcomes (g = 0.579, 95% CI = 0.131–1.027).

### Meta-Regression Analyses

Meta-regression analyses were performed for intervention length, duration, and frequency. When intervention length, duration, and frequency were used as covariates in the meta-regression analyses, the results were not significant (Supplementary Table 2).

## Discussion and Implications

This study investigated the effectiveness of robot care in the neuropsychiatric symptoms and social health of individuals with dementia. A meta-analysis was conducted to systematically evaluate the efficacy of robot care intervention and maintenance effect for people with dementia. A total of 1,672 people with dementia participated in the 20 studies reviewed in this meta-analysis, and the effect sizes were examined according to the moderators. Among the 20 studies included, seven reported sustained results of robotic interventions. The findings of this study provide evidence that robot care intervention and maintenance are beneficial for the social health of individuals with dementia.

### Major Findings

The overall effect size of intervention (*g* = 0.286) was similar to that reported for the behavioral and psychological symptoms of dementia (*g* = −0.38), such as agitation, depression, and neuropsychiatric symptoms by Leng et al. (2019). Lu et al. (2021) asserted that robot care could be a valuable asset in group treatment modalities or facilitate interactions among individuals with dementia, their caregivers, and therapists.

The findings revealed a statistically significant and small effect of robotic care intervention on the neuropsychiatric (*g* = 0.258), appraisal (*g* = 0.208), and social participation (*g* = 0.287) aspects in patients with dementia. Previous studies have identified several key mechanisms underlying the effectiveness of robotic interventions. First, robots act as social interaction enhancers, providing human-like stimulation through dialogue, emotional expressions, and simple interactions that help alleviate isolation in patients with dementia (Liang et al., 2017). Second, robots offer emotional support, inducing feelings of calm and safety, which can have a sedative effect and serve as an alternative to pharmaceutical treatment (Gustafsson et al., 2015). Third, robots encourage physical activity by suggesting walking exercises, stretching, or exercise programs to help prevent physical deterioration caused by inactivity (Khosla et al., 2021; Moyle et al., 2017). Additionally, robots assist in maintaining routines, reminding patients to take medication or eat at scheduled times, which is especially helpful for those with a disoriented sense of time (Jøranson et al., 2016; Moyle et al., 2017). Saragih et al. (2021) reported nonsignificant effect sizes for agitation, anxiety, cognitive function, depression, neuropsychiatric symptoms, total daytime sleep duration, and quality of life in 10 out of the 15 analyzed articles overlapping with the current study. Despite including the articles analyzed in the current study, the studies by Leng et al. (2019) and Lu et al. (2021) reported nonsignificant effect sizes of robotic interventions on cognitive function, quality of life, and agitation in people with dementia. Although previous meta-analyses have reported conflicting results, a direct comparison of the effect sizes derived from previous studies with the results of the current study is challenging for several reasons. First, the 20 primary studies analyzed in this study overlapped with the 10 studies examined by Saragih et al. (2021), 7 studies examined by Lu et al. (2021), and 6 studies examined by Leng et al. (2019). However, the current meta-analysis examined six more significant effect sizes than the study by Saragih et al. (2021) and eight more significant effect sizes than the studies by Leng et al. (2019) and Lu et al. (2021). Second, this study employed a shifting unit of analysis approach when conducting the overall and subgroup effect size analyses. In a meta-analysis, individual studies are assumed to be independent; however, when a single study reports multiple effect sizes, this assumption is violated. To address the violation of independence assumption when calculating the overall effect size, we treated each study and each subgroup effect size in the subgroup analyses as a single unit (Cooper et al., 2009). Previous meta-analyses lack information regarding the units of the subgroup analyses. In the current study, the significant effect size of robot care indicated its potential to enhance the social health of individuals with dementia.

Furthermore, this study explored the effect of robot care intervention maintenance reported in 7 of the 20 papers. Kim and Park (2017) performed a meta-analysis that revealed that person-centered care interventions conducted directly with patients with dementia yielded beneficial effects, albeit predominantly in the short term. This result underscores the significance of considering not only the transient but also the enduring effects of intervention. However, there are no previous meta-analyses of on the maintenance effect of robot care intervention for dementia. All seven studies investigated the maintenance of PARO’s intervention and revealed a significant effect (*g* = 0.279). These findings confirm the enduring efficacy of PARO in improving neurological cognition and alleviating psychosocial symptoms in people with dementia. The verification of the maintenance effect of robot care intervention represents a novel discovery regarding the therapeutic potential of robot care. Despite the relative inaccessibility of robot care to the general public owing to its high cost, the maintenance of its intervention effect can counterbalance these financial constraints, rendering it a highly appealing treatment modality for medical consumers. Notably, Mervin et al. (2018) underscored the cost-effectiveness of PARO’s social and psychological interventions for agitation by estimating their monetary value. The long-term benefits of robot care hold promise for its utility as a nonpharmacological treatment approach for dementia care.

In addition to PARO, we examined the impact of humanoid robots and Bomy. Among the 20 articles analyzed, 16 focused on the effects of PARO, 3 examined the effects of humanoid robots, and 1 investigated Bomy. Diverging from PARO and humanoid robots, Bomy resembles a video camera. However, its effects were not statistically significant. Conversely, PARO and humanoid robots exhibited statistically significant effects, with humanoid robots demonstrating a marginally higher impact than PARO. This discrepancy suggests a discernible difference in effectiveness based on robot type. Previous studies have indicated a preference for humanoid robots among older adults because of the belief that human-like features facilitate more natural interactions than the mechanistic visage of robots (Chu et al., 2019). However, a recent systematic review revealed that individuals with dementia exhibit limited comprehension of innovative technologies and a low level of interest, along with negative attitudes toward robots. This trend was evident even when compared with that for alternative technologies such as mobile technology, environmental sensors, and wearable devices (Hebesberger, Dondrup, Gisinger, & Hanheide, 2017). Therefore, in providing effective robot care for individuals with dementia, considerations must include the appearance and interface of robots, shaping them into a form that exudes friendliness and accessibility, which benefits individuals with dementia.

In this study, we confirmed significant intervention and maintenance effects of robot care for individuals with dementia on their neuropsychiatric symptoms and social health. No difference was found in the intervention effect size according to robot type. Although the effect size was small (*k* = 2), the effect size of maintenance on appraisal outcomes was 0.579, which must be confirmed through future research.

The significance of this study can be described as follows: First, the results of this study strengthen the application of nondrug therapies for dementia treatment. Given the risks associated with pharmacological treatments for anxiety and cognitive decline (e.g., side effects and limited efficacy), robotic interventions may offer safer and more sustainable alternatives. Second, robotic interventions, particularly using PARO robots, have been shown to produce lasting benefits over time. This suggests that robotic care can be effectively integrated into long-term care plans for people with dementia, thereby continuously improving their quality of life.

The implications of this study are as follows. First, the findings of this study support the integration of robots, such as PARO and humanoid robots, into clinical settings, such as nursing homes. The ability of these robots to alleviate neuropsychiatric symptoms while promoting social engagement makes them a viable treatment option, particularly for facilities seeking alternatives to reduce agitation and enhance patient interactions. Second, although robots can be expensive, the sustained effects identified in this study may offset initial costs, making robotic therapy financially more attractive for long-term dementia care. Finally, the findings of this study highlighted the need for further research on different robot types and their relative efficacies. The differences in effectiveness observed between humanoid robots and others, such as Bomy, suggest that robot design (e.g., human-like appearance) plays a crucial role in effectiveness, thus offering a direction for the development of more user-friendly, emotionally engaging robots tailored to people with dementia.

The limitations of this study are as follows. First, although this study analyzed 20 RCT articles, the number of papers analyzing the effect sizes of maintenance was small (*n* = 7). Regarding the maintenance effect, only the effect size by outcome could be analyzed, and no results indicated whether effect sizes varied with robot type. After accumulating data on the maintenance effect of robot care using humanoids, a necessary step is to determine whether such effect is significant. Second, we only determined the effects of robot care on appraisal and social participation. Outcomes for other social health aspects must also be analyzed. Third, this study found a significant effect size; however, further research is necessary to investigate the moderators that may influence the effect. In this study, the difference in effect sizes according to robot type and the dependent variable was investigated through a subgroup analysis to find a moderator; however, such a moderator was not found. As information on moderators is needed to implement effective interventions, future research on this topic is necessary.

## Supplementary Material

igae110_suppl_Supplementary_Sections_1_Figures_1-2_Tables_1-2

## Data Availability

The dataset supporting the conclusions of this study is available from the OSF repository at https://osf.io/xctru/.
