# Supplementary material for: Effectiveness of Robot Care Intervention and Maintenance for People with Dementia: A Systematic Review and Meta-Analysis
Source: Innov Aging. 2024 Dec 21;9(3):igae110. doi: 10.1093/geroni/igae110 (PMC11887035; doi:10.1093/geroni/igae110)
Supplement: igae110_suppl_Supplementary_Sections_1_Figures_1-2_Tables_1-2 [file igae110_suppl_supplementary_sections_1_figures_1-2_tables_1-2.docx]

***Innovation in Aging* Supplementary Material: Nam & Park. Effectiveness of Robot Care Intervention and Maintenance for People with Dementia: A Systematic Review and Meta-Analysis.**

Section 1.

Within the population, the keywords of “Alzheimer*,” “Cognitive Dysfunction,” “dement*,” or “mild cognitive impairment*)” were searched. Within the independent variable, the following keywords were searched: assistive technolog* OR augmented realit* OR blended OR computer-assisted OR computer-based OR digital* or ehealth OR E-health OR electronic-app* OR exergam* OR handheld-computer* OR hand-held-computer* OR hand-held-device OR handheld-device* OR Information Technology OR Internet OR iPad* OR iPhone* OR laptop* OR mhealth OR m-health OR microcomputer* OR mobile app* OR Mobile device* OR mobile health OR mobile phone* OR palmtop* OR PDA OR personal digital assist* OR remote consultation* OR Robot* OR smart phone* OR smartphone* OR software-app* OR Tablet OR teleconsultation* OR telemedicine* OR

telephone application* OR telepsychiatry OR telepsychology OR telerehabilitation* OR touchscreen* OR touch-screen* OR virtual realit* OR wearable* OR Web-based. Within the dependent variable, the following keywords were searched: “(activiti* OR adaptation OR autonomy OR community engagement OR community* OR empowerment OR experienced Autonomy OR friend* OR happiness OR isolation* OR lonel* OR mastery OR occupational function* OR overcome OR personal Autonomy OR potentials OR psychosocial OR quality of life OR resilience OR self-care skill* OR self-determination OR Self-efficacy OR Self-management OR Social activit* OR social capital OR social contact* OR social Determinants of Health OR social inclusion OR social isolation OR social network OR social obligation* OR social participation OR wellbeing OR well-being OR wellness.

Section 2.

Supplementary Figure 1. Flow Chart of Study Selection Process

Records identified through database search
(*n* = 2,325)

Additional records identified through literature review
(*n* = 13)

Remaining records after removing 741 duplicates (*n* = 1,584)

Records screened for eligibility (*n* = 1,584)

Title and abstracts excluded

(*n* = 1,300)

- Not related
- Not dementia
- No intervention
- Not group design

Full-text articles assessed for eligibility
(*n* = 284)

Records were excluded based on full-text review (*n* = 266)

- Not dementia (*n* = 51)
- No intervention (*n* = 50)
- Not RCT (*n* = 6)
- No data (*n* = 42)
- Not robot care (*n* = 117)

Studies included in qualitative synthesis and meta-analysis
(*n* = 20)

Full-text articles after cross-referencing the reference (*n* = 2)

Screening

Eligibility

Included

Identification

Supplementary Table 1. Overall effect sizes of intervention and maintenance

| Category | *n* | -95% *CI* | ES | +95% *CI* | SE | *Q* | *p* | *I*^2^ |
| --- | --- | --- | --- | --- | --- | --- | --- | --- |
| Intervention | 20 | 0.231 | 0.286 | 0.341 | 0.028 | 34.694 | 0.02 | 45.24 |
| Maintenance | 7 | 0.210 | 0.279 | 0.347 | 0.035 | 5.851 | 0.44 | 0.00 |

*Note. n =* number of studies; *CI* = confidence interval; ES = effect size; SE *=* standard error.

Supplementary Figure 2. Forest plot of robot care intervention maintenance


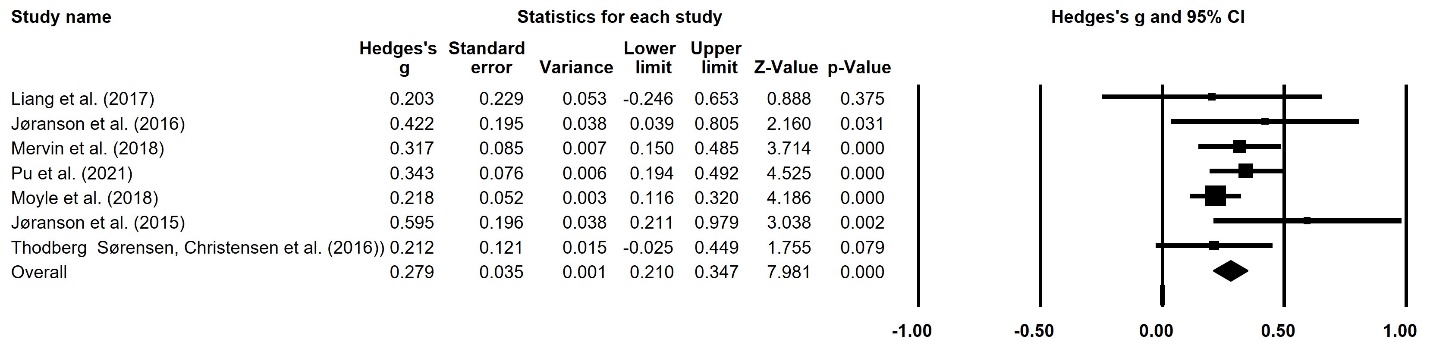


Supplementary Table 2. Effect size by continuous variables

| Continuous variable | $\beta$ | -95% *CI* | +95% *CI* | SE | *p* | *Q* | *R*^2^ |
| --- | --- | --- | --- | --- | --- | --- | --- |
| Intercept | 0.489 | -0.022 | 0.956 | 0.238 | 0.040 | 1.67 | 0.00 |
| Length | 0.000 | -0.009 | 0.009 | 0.005 | 0.924 |  |  |
| Duration | -0.002 | -0.008 | 0.004 | 0.003 | 0.577 |  |  |
| Frequency | -0.001 | -0.081 | 0.079 | 0.041 | 0.975 |  |  |

*Note. CI* = confidence interval; SE *=* standard error.
